# LC-MS-Based Metabolomic Approach Revealed the Significantly Different Metabolic Profiles of Five Commercial Truffle Species

**DOI:** 10.3389/fmicb.2019.02227

**Published:** 2019-09-25

**Authors:** Xiaolin Li, Xiaoping Zhang, Lei Ye, Zongjing Kang, Dinghong Jia, Lufang Yang, Bo Zhang

**Affiliations:** ^1^Soil and Fertilizer Institute, Sichuan Academy of Agricultural Sciences, Chengdu, China; ^2^Department of Microbiology, College of Resources, Sichuan Agricultural University, Chengdu, China

**Keywords:** truffles, metabolomics, LC-MS, bioactivity, edible fungus

## Abstract

Truffles are ascomycetous ectomycorrhizal fungi that have elevated status in the culinary field due to their unique aroma and taste as well as their nutritional value and potential biological activities. *Tuber melanosporum*, *T. indicum*, *T. panzhihuanense*, *T. sinoaestivum*, and *T. pseudoexcavatum* are five commercial truffle species mainly distributed in Europe or China. In this study, an untargeted metabolomics technology based on an ultra-high-performance liquid chromatography-tandem mass spectrometry (UHPLC-MS/MS) method was applied to analyze the metabolic profiles and variations among these five truffle species. In our results, a total of 2376 metabolites were identified under positive ion mode, of which 1282 had significantly differential amounts and covered 110 pathways or metabolisms. Principal component analysis (PCA) and partial least squares-discriminant analysis (PLS-DA) revealed a clear separation from each of these five truffles, indicating a significantly different metabolic profile among them, with the biggest difference between *T. melanosporum* and the other four truffles. The differential metabolites covered various chemical categories, and a detailed analysis was performed for nine metabolic categories, including amino acids, saccharides and nucleosides, organic acids, alkaloids, flavonoids, carnitines, phenols and alcohols, esters, and sulfur compounds. For each of the nine categories, most of metabolites predominantly accumulated in *T. melanosporum* compared with the other four truffles. Meanwhile, there were significant differences of the average ion intensity in each category among the five truffles, e.g., higher amounts of amino acids was detected in *T. panzhihuanense* and *T. pseudoexcavatum; T. indicum* contained significantly more carnitines, while there were more alkaloids in *T. melanosporum.* Additionally, some metabolites with biological activities were discussed for each category, such as acetyl-L-carnitine, adenine, neobavaisoflavone, and anandamide. Generally, this study may provide the valuable information regarding the variation of the metabolic composition of these five commercial truffle species, and the biological significance of these metabolites was uncovered to explore the metabolic mechanisms of truffles, which would be helpful for further research on the compounds and potential biological functions in truffles that have not yet been investigated.

## Introduction

*Tuber* spp., belonging to Ascomycota, are an ectomycorrhizal fungus that lives symbiotically with plants and is characterized by hypogeous fruiting bodies (Mello et al., 2006; Benucci and Bonito, 2016). Their fruiting bodies are also called truffles, and are referred to as “underground gold” because they are rare and are highly valued for their culinary and medicinal traits (Tang et al., 2015). At present, there are at least 180–230 truffle species that have been discovered throughout the world (Kues and Martin, 2011). *Tuber melanosporum* is the most highly appreciated black truffle due to its unique and intense fragrance. It is mainly produced in countries along the Mediterranean coast, such as Italy, France, and Spain, and commands a very expensive price in the edible fungus market (Bertault et al., 1998; Campo et al., 2017). *Tuber indicum* is also a black truffle species that is phylogenetically and morphologically close or similar to *T. melanosporum*, and it is one of the major commercial truffles in China (Zhang et al., 2019). In addition to *T. indicum, Tuber sinoaestivum*, and *Tuber pseudoexcavatum* are all Chinese black truffles with commercial value, and they have been exported to Europe, the United States, and Australia, although they do not command a price as high as *T. melanosporum* (García-Montero et al., 2010; Zambonelli, 2012; Ye et al., 2018). Also, the investigations about the *T. sinoaestivum* and *T. pseudoexcavatum* were relatively rare compared with *T. melanosporum* and *T. indicum. Tuber panzhihuanense*, also called the Chinese white truffle, is a unique truffle species in southwest China discovered in 2011 with a pleasant aroma and flavor, and is also the only white truffle species with great commercial potential found in China (3000–5000 RMB/kg market value) (Deng et al., 2013; Yang et al., 2019).

In addition to the unique and seductive aroma of truffles, the nutrients and bioactive compounds with health benefits in truffles are also the major attraction, leading to a series of investigations to explore the metabolites in their fruiting bodies. It has been found that truffles are rich in protein, fatty acids, carbohydrates, amino acids, and minerals (Wang and Marcone, 2011; El Enshasy et al., 2013). Some important metabolites with biological activities have been isolated and characterized from truffle fruiting bodies, including polysaccharides, androstenol, ceramides, ergosterol, and phenolics, which have been verified to possess multiple healthy attributes (Zhao et al., 2014; Longo et al., 2017; Patel et al., 2017). For example, polysaccharide extracts and ribonuclease from truffle fruiting bodies showed high antioxidant potential, immunomodulatory and antitumor activities (Zhao et al., 2014). In addition, anti-inflammation, antimicrobial, anti-depressant, and menstruation regulation properties in truffles have also been also demonstrated (Wang and Marcone, 2011). Furthermore, the chemical compounds contributing to the aroma of truffles have been studied to explore the secrets of truffles fragrance, which is closely related to the microbiome in truffles, and over 200 volatile organic compounds (VOCs) have been isolated and reported (Zhang et al., 2016). The aroma of truffles is finally determined by the comprehensive action of various compounds, including alcohols, ketones, enols, aldehydes, esters, aromatic compounds, amines, and sulfur compounds (Mannina et al., 2012; Splivallo and Ebeler, 2015). However, the nutrients, bioactive compounds, and VOCs are variable in different truffle species, meaning that there are different metabolite profiles in different truffle species.

Metabolomics is a newly developed technique in the field of systematic biology following genomics and proteomics, and it explores the metabolic mechanism of the entire organism by detecting the changes in metabolites (Fiehn et al., 2000; Zhao et al., 2019). Metabolites provide a functional output of biochemical activity as the downstream of genes, transcripts, and proteins (Patti, 2011). Currently, metabolomics has been utilized in medicine and botany, and for the analysis of food quality, nutrition, and components. An untargeted metabolomics analysis simultaneously detects as many metabolites as possible, to systematically compare the features of metabolites among species, and is also suitable for truffles (Zhao et al., 2019). Many studies have reported the metabolites in different truffle fruiting bodies under different conditions, with the methods of standard wet chemistry analysis, spectrophotometry, gas chromatography-olfactometry (GC/O), gas chromatography-mass spectrometry (GC-MS), NMR spectroscopy, picot-electronic nose, and liquid chromatography-mass spectrometry (LC-MS), to detailly reveal the compositions of the chemical compounds (Wang and Marcone, 2011; Patel et al., 2017). However, many of these previous studies were more concerned about how to extend the shelf life of fresh truffles by detecting some of the metabolite components that contributed to truffle commercial use, or analyzed the aroma of changing VOCs without the entire metabolic profile or the metabolic mechanism of truffle fruiting bodies. For example, Savini et al. (2017) only detected the phenolic profiles and volatile profile of *T. melanosporum* by high-performance liquid chromatography-high resolution mass spectrometry (HPLC-HRMS) under different packaging conditions. Additionally, these studies focused more on well-known truffle species, such as *Tuber magnatum*, *T. melanosporum*, *Tuber borchii*, and *Tuber aestivum*, and rarely provided the details regarding Chinese truffle species, which limits the further exploration of the chemical compounds and the potential biological functions of truffles.

In this study, an LC-MS-based metabolomic approach was used to investigate the metabolic composition and variations among five commercial truffle species, including *T. melanosporum* and four Chinese truffles, aiming to reveal the differences in the metabolic profile and compare their edible values. In addition, the biological significance of these metabolites was uncovered to explore the metabolic mechanisms of truffles, which would be helpful to find out the formation mechanism of truffle ascocarps, which thus far has been unclear. To the best of our knowledge, this is the first study that used metabolomics to enhance the insight into the metabolic variation of these five truffle species.

## Materials and Methods

### Strategy for Sampling Truffle Fruiting Bodies and Associated Soils

**FIGURE 1:**
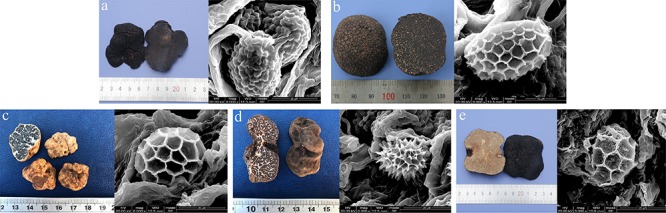
Images of one of the ascocarps and their mature spores of each truffle species used for LC-MS-based metabolomic analysis. **(a)** *T. melanosporum*, **(b)** *T. indicum*, **(c)** *T. panzhihuanense*, **(d)** *T. pseudoexcavatum*, and **(e)** *T. sinoaestivum.* The spores were observed under the scanning electron microscope (SEM) (Inspect, FEI, United States) and these ascocarps were taxonomically identified by morphological and molecular methods at the Soil and Fertilizer Institute, Sichuan Academy of Agricultural Sciences.

There were five commercial truffle species selected for this study: *T. melanosporum*, *T. indicum*, *T. panzhihuanense*, *T. sinoaestivum*, and *T. pseudoexcavatum* (Figure 1). The fruiting bodies from these *Tuber* spp. were taxonomically identified by morphological method and molecular method based on ITS sequence amplified with primers ITS1F (5′-CTTGGTCATTTAGAGGAAGTAA-3′) and ITS4 (5′-TCTCGGGCTGGAGGTGCGGGTCGAGT-3′). The nucleotides alignment of the obtained sequences was performed based on the National Center for Biotechnology Information (NCBI) database and the phylogenetic analysis of these five truffle species was also carried out by Mega 6.0 (Supplementary Figure S1). The nucleotide sequences in this study have been deposited in NCBI database with GenBank accession numbers MN338092–MN338096.

The sampling locations and the corresponding host plants for these different truffle species are shown in Table 1. For each truffle species, a total of thirty ascocarps from close geographical position and at the same maturity were collected. These ascocarps were all at the completely maturity stage and the maturation degrees were judged by the ascospores, peridium, gleba, size and weight according to Le Tacon et al. (2013) and Antony-Babu et al. (2014). When collecting in the field, the ascocarps were removed from the ground by hand. Then, the unbroken ascocarps were selected, wrapped with aluminum foil, and placed in a low-temperature storage box with ice packs, after which they were immediately transported to the laboratory. Meanwhile, the surrounding soils of the ascocarps in five different sampling locations were also collected for soil properties analysis, which were stored at 4°C. There were three biological replicates of associated soil samples from each truffle species.

**TABLE 1:** The sampling locations and host plants of five commercial truffle species in this study.

**Sample ID**	***Tuber* spp.**	**Sampling locations**	**Host plants**
mel	*Tuber melanosporum*	Cahors, Midi-Pyrénées Region, France	*Quercus pubescens*
ind	*Tuber indicum*	Dayao county, Chuxiong Yi Autonomous Prefecture, Yunnan, China	*Pinus yunnanensis*
pan	*Tuber panzhihuanense*	Renhe county, Panzhihua city, Sichuan, China	*Pinus yunnanensis*
sin	*Tuber sinoaestivum*	Dayao county, Chuxiong Yi Autonomous Prefecture, Yunnan, China	*Pinus yunnanensis*
pse	*Tuber pseudoexcavatum*	Huidong county, Liangshan Yi Autonomous Prefecture, Sichuan, China	*Pinus armandii*

*One sample ID represents thirty ascocarps from close geographical position (with the range about 4 m^2^) and at the same maturity of the same truffle species.*

In the laboratory, the ascocarps were cleaned with small brushes to wipe off the soil, rinsed three times with sterile water, and then surface-disinfected with 75% alcohol. Broke the ascocarps and then carved a piece on their inside with the sterile blade on a clean bench, and their tissues were transferred with sterile tweezers into 5 ml sterile Eppendorf tubes. The tubes were placed into liquid nitrogen for 30 s, and then quickly stored at –80°C until analysis. In order to reduce error, tissues from three ascocarps (the same truffle species) were mixed into one biological sample, so there were ten biological samples obtained from thirty ascocarps of each truffle species, which finally underwent metabolic analysis. No less than 100 mg fruiting bodies per sample were prepared for metabolite extraction. These samples from five *Tuber* species were assigned as “mel,” “sin,” “ind,” “pan,” and “pse.”

### Soil Properties Analysis

The properties of the associated soil samples from five different sampling locations were analyzed according to the previously described method (Li et al., 2016), including pH, organic matter content (OM), total nitrogen (TN), available nitrogen (AN), available phosphorus (AP) and available potassium (AK). Briefly, soil pH was determined with a soil–water (1:5, w/v) slurry using a compound electrode; OM was measured using the Tyurin method; TN was determined by the Kjeldahl method; AN was measured with the alkali solution diffusion method; AP was extracted with NaHCO_3_ and determined by the ammonium molybdate ascorbic method; and ammonium acetate extraction – flame photometry was used to measure AK.

### Metabolite Extraction

The process of metabolite extraction for ultra-high performance liquid chromatography-tandem mass spectrometry (UHPLC-MS/MS) was as follows, according to previous methods (De Vos et al., 2007; Li et al., 2018). Use the frozen samples to ground into fine powders with liquid nitrogen, and then a total of 100 mg powder was weighed in centrifuge tubes. Afterward, 400 μL 80% (v/v) methanol aqueous solution (pre-cooled at –20°C) was added, followed by vortexing and oscillating for 30 s (Vortex Mixer, QL-866). The homogenates were subsequently placed at –20°C for 60 min and centrifuged at 14,000 × *g*, at 4°C for 20 min (Thermo Fisher Scientific, ST16R). All the supernatant was then transferred into 1.5-mL Eppendorf tubes, frozen, and dried under vacuum (Labogene, Scan Speed 40). The residue was dissolved with 100 μl complex solvents, vortexed and oscillated again, and centrifuged at 14,000 × *g*, at 4°C for 15 min. Finally, the supernatant was purified by passing through a 0.22-μm membrane filter for LC-MS detection. To monitor and evaluate the system stability throughout the experiment, equal amounts of supernatant from each processed sample were mixed as quality control (QC) samples. Meanwhile, take the blank matrix of the experimental sample as the blank samples, which composed of the reagents used before and were equally treated to the experimental samples. Blank samples also underwent analysis to remove the background ions.

### UHPLC-MS/MS Analysis for Untargeted Metabolomics

The chromatographic separation was performed using an Accucore HILIC column (50 mm × 2.1 mm, 2.6 μm, Thermo Fisher Scientific^TM^, United States) fitted to the Thermo Scientific Vanquish UHPLC system. Metabolites were eluted from the column using a gradient mobile phase that consisted of phase A (0.1% formic acid, 10 mM ammonium acetate, and 95% acetonitrile) and phase B (0.1% formic acid, 10 mM ammonium acetate, and 50% acetonitrile), at a flow rate of 0.3 mL/min. A volume of 5 μL per sample was injected after equilibration. The temperature of the column and auto-sampler was maintained at 40 and 4°C, respectively. The linear gradient elution procedure was set as follows: 2% B for 0–1 min, from 2% B at 1 min to 50% B at 17 min until 17.5 min, then returning to the initial gradient conditions (2% B) at 18 min, followed by maintaining at 2% B for 18–20 min. In order to avoid the influence caused by the detected signal fluctuation of the apparatus, samples were randomly injected. A QC sample followed by a blank sample was injected after every five experimental sample injections.

The electrospray ionization-mass spectrometry (ESI-MS) experiments were carried out using a Thermo Q Exactive^TM^ HF-X mass spectrometer with a spray voltage of 3.2 kV in both positive and negative modes. The sheath gas and auxiliary gas flow rate were set at 35 and 10 arbitrary units, respectively. The capillary temperature was 320°C. The MS analysis alternated between MS full scans and data-dependent MS/MS scans with dynamic exclusion, and the mass scan range was selected from 100–1500 *m*/*z* at a scan rate of 40 Hz.

### Data Acquisition and Processing

After the detection and analysis of truffle ascocarps by UHPLC-MS/MS methods, the total ion chromatograms of all the samples were extracted. The acquired raw MS files were processed with the Compound Discoverer (Thermo Fisher Scientific) software for data pretreatments including peak identification, peak alignment, peak feature extraction, and peak area normalization, running separately under positive and negative ionization mode (Ganna et al., 2015; Yu et al., 2018).

Firstly, screening for the retention time (RT), mass-to-charge ratio (*m*/*z*) and other parameters, and the peak alignment of different samples was conducted according to the retention time deviation of 0.2 min and the mass deviation of 5 ppm in order to make the identification more accurate. Then, the peak extraction was performed according to the set information and adduct information: mass deviation = 5 ppm, signal strength deviation = 30%, signal-to-noise ratio = 3, minimum signal strength = 100000. Additionally, the peak area was quantified. The mass spectrometry matrix data containing sample names, *m*/*z*-retention time pairs, and ion intensity information were generated and exported. The target ions were then integrated to predict the molecular formula and compared against ChemSpider and mzCloud online databases for the identification and confirmation of the compounds. The background ions were removed with blank samples, and the quantitative results were normalized with QC samples. Finally, the identification and quantitative results from the data were obtained and used for subsequent statistical analysis.

### Statistical Analysis

Multivariate statistical analysis of metabolites, including principal component analysis (PCA) and partial least squares-discriminant analysis (PLS-DA), were carried out by SIMCA-P software (v13.0, Umetrics, Umeå, Sweden) after Pareto scaling, in order to reveal the differences in the metabolic composition among the ascocarps of the five different *Tuber* species. The reliability of the PLS-DA model was verified by permutation test, which was used to evaluate whether the model was overfitted. An independent samples Kruskal–Wallis one-way analysis of variance (ANOVA) followed by the Dunn–Bonferroni *post hoc* method was used for the pairwise multiple comparisons of each metabolite (IBM^®^ SPSS^®^ v22). Additionally, variable importance in the projection (VIP) value of the first principal component in the PLS-DA model combined with fold change (FC) were also computed to screen out the significantly differential metabolites. The threshold was set as: VIP > 1, corrected *P* < 0.05, and FC > 2.0 or FC < 0.5. The volcano plots made by R package DESeq2 were used to analyze the differential log_2_-FC- and log_10_-P-transformed values of each metabolite. In addition, hierarchical clustering analysis (HCA) was performed with the pheatmap-package in R software (v3.3.2) to visualize the metabolite profiles and reveal the relationship between metabolites and samples. Furthermore, the biological significance of metabolites was uncovered through the functional analysis of metabolic pathways using the Kyoto Encyclopedia of Genes and Genomes (KEGG) pathway analysis. The KEGG database provides an excellent integrated metabolic pathway query and is helpful for metabolic analysis and metabolic network research (Kanehisa, 2016). The hypergeometric test was used to determine the significantly enriched KEGG pathways of differential metabolites by comparison with all the identified metabolites.

Statistical analysis of the soil properties’ data was performed by one-way analysis of variance (ANOVA) using SPSS 22.0. Least significant difference (LSD) was used to test if the results between different treatments were significant at *P* < 0.05.

## Results

### The Differences of the Soil Properties Associated With Five Different Truffle Species

The properties of the soils from different truffle sampling locations were shown in Table 2, which reflected the significant differences in the basic characteristics of the soils around the five truffle species. The pH was significantly higher in the soil of *T. melanosporum* (*P* < 0.05), which presented alkaline, while the soils around the other four truffles were slightly acidic. The soil pH of *T. sinoaestivum* and *T. pseudoexcavatum* were significantly higher than that of *T. indicum* and *T. panzhihuanense* (*P* < 0.05). The OM content of the soils around the five truffles is significantly different from each other (*P* < 0.05), with the highest OM content in the soil of *T. sinoaestivum* and the lowest in *T. panzhihuanense*. The soil associated with *T. pseudoexcavatum* showed the highest TN content, followed by *T. indicum* (*P* < 0.05). However, the AN content was highest in the soil of *T. melanosporum*, also followed by *T. indicum* (*P* < 0.05). The content of both TN and AN were the lowest in the soil of *T. panzhihuanense.* There was significantly more AP in the soil of *T. sinoaestivum* and significantly more AK in the soil of *T. indicum* and *T. panzhihuanense* compared with the other soil samples (*P* < 0.05).

**TABLE 2:** The properties of the soil from different truffle sampling locations.

**Sample ID**	**pH**	**OM g/kg**	**TN g/kg**	**AN mg/kg**	**AP mg/kg**	**AK mg/kg**
mel	7.99 ± 0.20a	36.60 ± 1.56d	2.37 ± 0.14d	181.00 ± 4.58a	23.60 ± 1.91c	139.33 ± 14.05d
ind	5.54 ± 0.21c	52.80 ± 2.31b	4.48 ± 0.20b	147.33 ± 6.81b	33.53 ± 1.45b	325.33 ± 7.02a
pan	5.49 ± 0.13c	26.30 ± 2.25e	1.21 ± 0.31e	106.33 ± 18.58d	11.53 ± 1.40d	309.00 ± 10.82a
sin	6.25 ± 0.07b	62.43 ± 2.70a	3.78 ± 0.21c	121.00 ± 3.60cd	43.40 ± 0.82a	270.67 ± 13.20b
pse	6.50 ± 0.25b	40.90 ± 1.49c	5.63 ± 0.38a	125.67 ± 6.03c	31.20 ± 1.54b	223.67 ± 7.77c

*ind, *Tuber indicum*; mel, *Tuber melanosporum*; pan, *Tuber panzhihuanense*; sin, *Tuber sinoaestivum*; pse, *Tuber pseudoexcavatum*. Each value is the mean of 3 replicates (±SD). Values followed by different lowercase letters indicate significant differences (*P* < 0.05) between samples in a line.*

### Untargeted Metabolomic Profiling of Five Truffle Species – Quality Control and Metabolite Quantitative Analysis

The correlation of QC samples indicated that the system stability and the quality of the acquired data were fine (Supplementary Figure S2), and the correlation coefficient (*R*^2^) of QC samples in positive ion mode was higher than that in negative ion mode. Additionally, the PCA plots of all samples showed that the distribution of QC samples was clustered together (Supplementary Figure S3), which also indicated satisfactory data quality.

**FIGURE 2:**
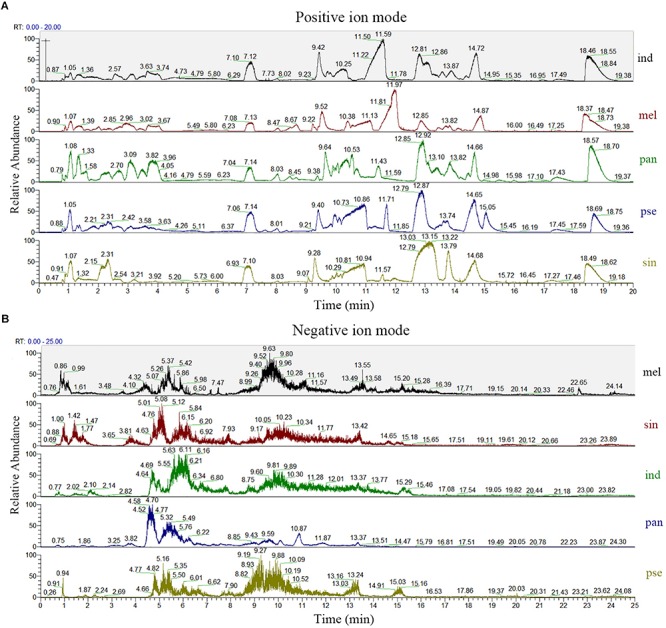
The total ion chromatogram of the five truffle species in both positive **(A)** and negative **(B)** ion modes. Abbreviations: ind, *Tuber indicum*; mel, *Tuber melanosporum*; pan, *Tuber panzhihuanense*; sin, *Tuber sinoaestivum*; pse, *Tuber pseudoexcavatum*.

A comparison of the total ion chromatograms of the extracts (Figure 2) showed that the types of metabolites in the ascocarps of five truffle species were similar, but the content of many metabolites was different. From the five *Tuber* species, a total of 7278 and 2460 metabolites were detected under positive and negative ion mode, respectively, of which 2376 and 843 named metabolites in positive and negative ion mode were finally identified and confirmed, respectively. The total ion intensity of all the identified metabolites in the five different truffle species is shown in a violin plot (Supplementary Figure S4), which reflects the intensity and density distribution of each metabolite in different groups at the overall level. It was found that the metabolite intensity of each truffle species was obviously higher under positive ion mode compared with negative ion mode, and the metabolites of *T. melanosporum* exhibited greater average ion intensity than other truffle species under positive ion modes. The distribution range of metabolite intensity for each truffle species was similar in positive ion mode.

According to the results reported above, because more metabolites were identified and data of higher quality were acquired in positive ion mode, the subsequent analysis was carried out under this mode.

### The Metabolic Variations Among the Five Truffle Species

**FIGURE 3:**
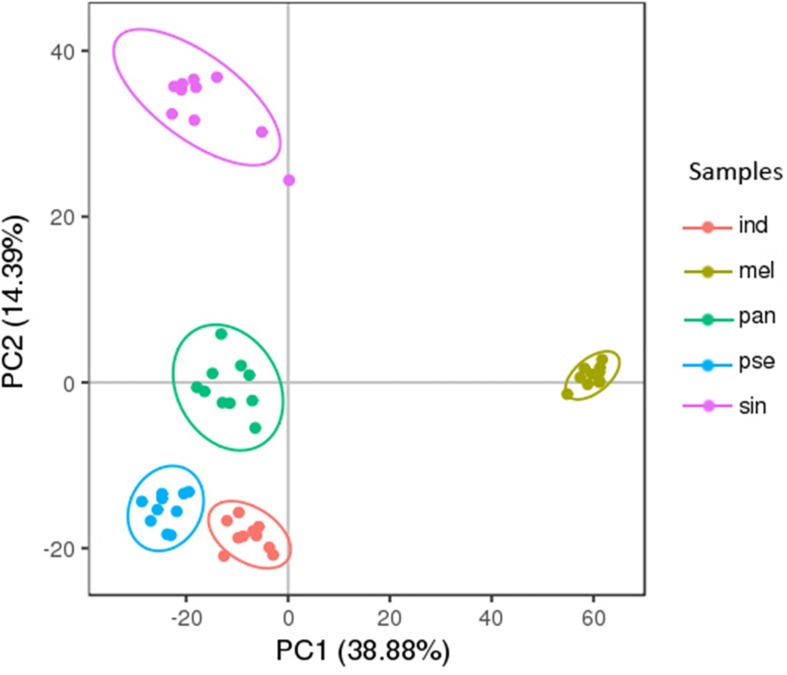
The principal component analysis (PCA) score plots of the five truffle species in positive ion mode. Abbreviations: ind, *Tuber indicum*; mel, *Tuber melanosporum*; pan, *Tuber panzhihuanense*; sin, *Tuber sinoaestivum*; pse, *Tuber pseudoexcavatum*.

The PC analysis of the 2376 metabolites (except for the QC samples) was displayed in Figure 3. The first two principal components of the PCA score plot were responsible for 53.27% (38.88% for PC1 and 14.39% for PC2) of the overall variance of the metabolite profiles, showing a clear separation of these five truffle species. Therefore, the metabolite profiles of the five truffle species were different from each other. There was a great degree of separation between *T. melanosporum* and the other four truffle species, indicating that the metabolic profiles of *T. melanosporum* were prominently different from those of the other four truffles. Additionally, *T. melanosporum* was separated from the other species mainly along PC1. However, the differences in the metabolic profiles among *T. indicum*, *T. panzhihuanense*, and *T. pseudoexcavatum* were relatively small due to the closer distribution of the three truffle species in the PCA plot, and these three truffles were separated from *T. sinoaestivum* by a great distance along PC2. To further verify the difference in metabolic profiles among these truffles, PLS-DA was carried out, which showed a clear separation for pairwise comparison of the five truffles (Supplementary Figure S5). The validation of the PLS-DA models presented a high interpretation rate (*R*^2^Y) and prediction degree (*Q*^2^ > 0.5), indicating a higher reliability of these models (Supplementary Figure S6).

### Screening and Classification of the Differential Metabolites

**FIGURE 4:**
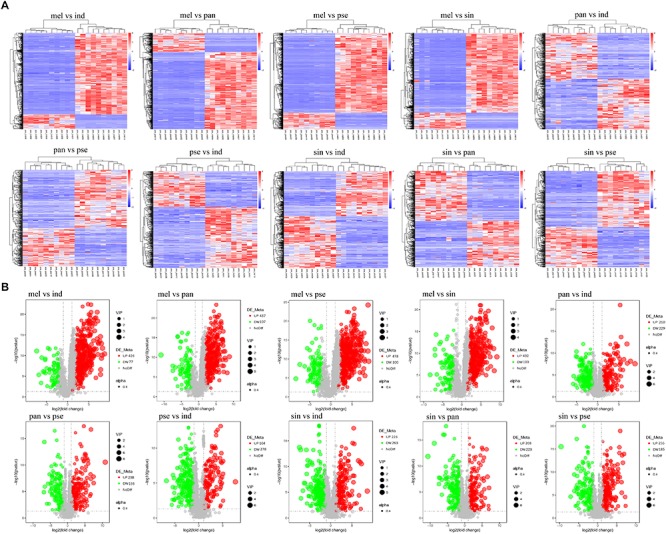
The **(A)** heatmaps and **(B)** volcanic plots of differential metabolites in pairwise comparison among the five truffle species. Abbreviations: ind, *Tuber indicum*; mel, *Tuber melanosporum*; pan, *Tuber panzhihuanense*; sin, *Tuber sinoaestivum*; pse, *Tuber pseudoexcavatum*.

A total of 1282 potential metabolites with significant differential amounts among the five truffle species were screened by statistical analysis, and the profiling overview of these differential metabolites in pairwise comparison of the five truffle species is shown in the heatmaps and volcano plots in Figure 4. The number of differential metabolites between *T. melanosporum* and *T. sinoaestivum* was the greatest, while the number of those between *T. panzhihuanense* and *T. pseudoexcavatum* was the smallest. There were clearly more upregulated differential metabolites for *T. melanosporum* when compared with the other four truffles, but there were more downregulated metabolites in *T. pseudoexcavatum* when compared with the others.

**FIGURE 5:**
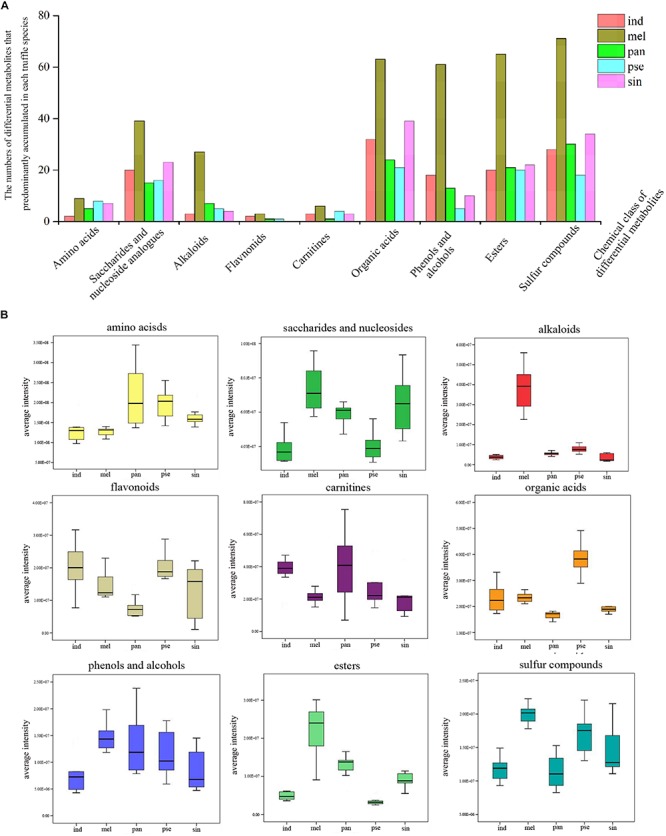
**(A)** The numbers of the differential metabolites with the greatest amounts in each truffle species compared with the other truffles and **(B)** the average ion intensity in each chemical class in the five truffle species. Abbreviations: ind, *Tuber indicum*; mel, *Tuber melanosporum*; pan, *Tuber panzhihuanense*; sin, *Tuber sinoaestivum*; pse, *Tuber pseudoexcavatum*.

All the 1282 differential metabolites were assigned to various chemical categories, including amino acids, saccharides, nucleosides and their analogs, organic acids, alkaloids, flavonoids, carnitines, and others. From another classification perspective, these metabolites covered amides, ketones, phenols and alcohols, esters, sulfur compounds, and others. The numbers of the differential metabolites that accumulated with the greatest frequency in each truffle species for some of these chemical categories were calculated (Figure 5A). In the nine selected categories shown in Figure 5A, compared with the other four truffles, the numbers of metabolites that predominantly accumulated in *T. melanosporum* were the most. Except for *T. melanosporum*, the numbers of saccharides and nucleosides, organic acids, esters, and sulfur compounds that accumulated with the greatest frequency in *T. sinoaestivum* were the maximum, and there was a greater number of phenols and alcohols accumulated most in *T. indicum* compared with the remaining three truffles, with the opposite occurring for amino acids and alkaloids. There were less predominantly accumulated organic acids, saccharides, and nucleosides in *T. panzhihuanense* and *T. pseudoexcavatum*.

### Differential Metabolite Analysis of the Five Truffle Species

The average ion intensities of the differential metabolites belonging to the nine selected categories are displayed in Figure 5B, and each category showed a significant difference among the five truffle species (*P* < 0.05). The important differential metabolites with pharmacological activities or with more attention before in each category are displayed in Table 3. Amino acids had the strongest intensities in *T. panzhihuanense* and *T. pseudoexcavatum*, and were significantly higher than that in *T. melanosporum* and *T. indicum* (*P* < 0.05). Among these differential amino acids, significantly more ornithine, asparagine, isoleucine, cysteine, and citrulline were detected in *T. sinoaestivum* (*P* < 0.05). Threonine showed significantly higher amounts in *T. pseudoexcavatum* (*P* < 0.05), and *T. melanosporum* contained more proline and leucine compared with the other truffles. There was more methionine and taurine in *T. indicum* and more valine in *T. panzhihuanense*, respectively.

**TABLE 3:** Some of the significantly differential metabolites among the five truffle species and their chemical categories^a^.

**Chemical class**	**Metabolites name**	**Formula**	**Molecular Weight**	**Retention time (min)**	**Ion intensity**	**Pairwise comparation with significant difference**	***P*-value^b^**
Amino acids	Ornithine	C_5_ H_12_ N_2_ O_2_	132.08982	13.695	sin > pan > ind > pse > mel	mel vs pan mel vs sin pse vs pan pse vs sin	0.000
	Asparagine	C_4_ H_8_ N_2_ O_3_	132.05337	10.109	sin > pse > ind > mel > pan	mel vs sin mel vs pse pse vs pan pan vs sin	0.000
	Isoleucine	C_6_ H_13_ N O_2_	131.09447	4.204	sin > mel > pan > pse > ind	sin vs ind sin vs pse sin vs pan	0.001
	D-(+)-Proline	C_5_ H_9_ N O_2_	115.06345	13.338	mel > sin > pse > pan > ind	mel vs ind mel vs pse mel vs pan	0.000
	Valine	C_5_ H_11_ N O_2_	117.0791	4.873	pan > ind > mel > pse > sin	sin vs ind sin vs pan pse vs pan pse vs ind	0.000
	Threonine	C_4_ H_9_ N O_3_	119.05831	9.899	pse > pan > ind > sin > mel	mel vs ind mel vs pse mel vs pan mel vs sin sin vs pse	0.000
	Cystine	C_6_ H_12_ N_2_ O_4_ S2	240.02338	14.679	sin > mel > pan > pse > ind	sin vs ind mel vs ind pan vs ind sin vs pse mel vs pse pan vs pse	0.000
	L-(+)-Citrulline	C_6_ H_13_ N_3_ O_3_	175.09542	11.381	sin > pse > ind > pan > mel	mel vs sin pan vs sin	0.000
	Methionine	C_5_ H_11_ N O_2_ S	149.05086	5.37	ind > mel > pan > pse > sin	sin vs ind sin vs mel sin vs pan ind vs pse mel vs pse pan vs pse	0.000
	Leucine	C_6_ H_13_ N O_2_	131.09455	10.093	mel > pan > sin > ind > pse	mel vs ind mel vs pse mel vs sin pan vs pse	0.000
	Taurine	C_2_ H_7_ N O_3_ S	125.01466	4.512	ind > mel > sin > pan > pse	mel vs pan mel vs pse ind vs pse ind vs pan	0.000
Saccharides and nucleosides	Adenine	C_5_ H_5_ N_5_	135.05409	2.6	sin > mel > pse > pan > ind	sin vs pan sin vs pse sin vs ind	0.000
	Adenosine	C_10_ H_13_ N_5_ O_4_	267.09621	2.251	sin > pse > pan > ind > mel	mel vs sin mel vs pse	0.001
	2′-Deoxyadenosine	C_10_ H_13_ N_5_ O_3_	251.10131	2.798	mel > sin > ind > pan > pse	mel vs pan mel vs pse mel vs ind sin vs pse	0.000
	Hypoxanthine	C_5_ H_4_ N_4_ O	136.03806	1.865	pse > ind > mel > pan > sin	pse vs sin pse vs pan	0.003
	5′-S-methyl-5′-thioadenosine	C_11_ H_15_ N_5_ O_3_ S	297.08871	1.763	ind > pan > mel > pse > sin	sin vs pan sin vs ind pse vs ind	0.000
	Nicotinamide ribotide	C_11_ H_15_ N_2_ O_8_ P	334.05598	16.341	pse > pan > ind > sin > mel	mel vs pan mel vs pse sin vs pan sin vs pse	0.000
	UDP-*N*-acetylglucosamine	C_17_ H_27_ N_3_ O17 P2	607.08025	13.338	pan > pse > ind > sin > mel	mel vs pan mel vs pse mel vs ind sin vs pan	0.000
	*N*-Acetyl-D-galactosamine	C_8_ H_15_ N O_6_	221.08944	9.855	mel > sin > ind > pan > pse	mel vs pan mel vs pse mel vs ind	0.000
	spectinomycin	C_14_ H_24_ N_2_ O_7_	332.15753	7.39	mel > ind > pse > pan > sin	sin vs ind sin vs mel sin vs pse mel vs pan pan vs ind	0.000
	Dihydrothymine	C_5_ H_8_ N_2_ O_2_	128.05849	9.805	ind > pse > sin > pan > mel	mel vs ind mel vs pse pan vs ind	0.000
	Glucose 1-phosphate	C_6_ H_13_ O_9_ P	260.02923	13.469	ind > pan > sin > pse > mel	mel vs pan mel vs pse mel vs ind mel vs sin	0.000
	Glucosylceramide	C_42_ H_79_ N O_8_	725.57836	4.94	pse > mel > pan > sin > ind	pse vs ind mel vs ind sin vs pse mel vs sin pan vs pse	0.000
	7-Methylguanosine	C_11_ H_15_ N_5_ O_5_	297.10666	2.219	pan > pse > mel > sin > ind	pse vs ind pan vs ind sin vs pse pan vs sin mel vs pan	0.000
Alkaloids	Agroclavine	C_16_ H_18_ N_2_	238.14647	2.322	mel > sin > pan > pse > ind	mel vs pan mel vs pse mel vs ind ind vs sin	0.000
	Sinapine	C_16_ H_23_ N O_5_	309.15488	0.996	ind >> mel > pan > sin > pse	pse vs ind mel vs pse sin vs ind mel vs sin pan vs ind	0.000
	Betaine	C_5_ H_11_ N O_2_	117.07903	13.044	mel > ind > pan > pse > sin	mel vs pan mel vs pse mel vs sin sin vs ind	0.000
	Leonurine	C_14_ H_21_ N_3_ O_5_	311.14746	10.938	mel > pse > sin > pan > ind	mel vs pan mel vs ind mel vs sin sin vs ind ind vs pse	0.000
	Colchicine	C_22_ H_25_ N O_6_	399.17446	10.926	sin > pse > mel > pan > ind	sin vs pan sin vs ind pse vs ind pse vs pan mel vs ind	0.000
	Piperlongumine	C_17_ H_19_ N O_5_	317.12505	1.023	mel > ind > pan > sin > pse	mel vs pan mel vs pse mel vs sin ind vs pse	0.000
	Sparteine	C_15_ H_26_ N_2_	234.20907	1.064	mel > ind > sin > pan > pse	mel vs pan mel vs pse mel vs sin	0.000
Flavonoids	6-Methylflavone	C_16_ H_12_ O_2_	236.08266	13.334	ind > pse > pan > mel > sin	mel vs pan pan vs ind sin vs ind	0.000
	2′-Methoxyflavone	C_16_ H_12_ O_3_	252.07743	10.417	pan > ind > sin > mel > pse	pse vs ind pan vs pse mel vs ind mel vs pan sin vs pan	0.000
	Kolaflavanone	C_31_ H_24_ O12	588.12749	4.508	ind > mel > pan > sin > pse	pse vs ind sin vs ind pan vs ind mel vs pan mel vs sin mel vs pse	0.000
	Neobavaisoflavone	C_20_ H_18_ O_4_	322.11909	9.622	mel > pan > ind > pse > sin	mel vs ind mel vs pse mel vs sin sin vs pan	0.000
Carnitines	Acetyl-L-carnitine	C_9_ H_17_ N O_4_	203.11522	9.416	ind > pan > sin > pse > mel	mel vs ind mel vs pan pse vs ind pse vs pan	0.000
	L(-)-Carnitine	C_7_ H_15_ N O_3_	161.10499	12.378	pse > sin > pan > ind > mel	mel vs pse mel vs sin pse vs ind sin vs ind	0.000
	(2E)-hexadecenoylcarnitine	C_23_ H_43_ N O_4_	397.31838	1.041	pse > ind > pan > mel > sin	sin vs pse sin vs ind sin vs pan mel vs pse mel vs ind	0.000
Organic acids	4-Pyridoxic acid	C_8_ H_9_ N O_4_	183.05289	1.567	ind > pan > mel > pse > sin	sin vs ind sin vs pan mel vs sin pse vs ind	0.000
	Nicotinic acid	C_6_ H_5_ N O_2_	123.03204	10.104	ind > pan > mel > pse > sin	sin vs ind sin vs pan mel vs sin pse vs ind pse vs pan	0.000
	Aspirin	C_9_ H_8_ O_4_	180.04195	3.98	pse > pan > mel > ind > sin	sin vs pse mel vs pse pse vs ind sin vs pan	0.000
	Mesalazine	C_7_ H_7_ N O_3_	153.04231	0.975	mel > sin > pse > ind > pan	mel vs pan mel vs pse mel vs ind pan vs sin ind vs sin	0.000
	(E)-Ferulic acid	C_10_ H_10_ O_4_	194.0575	4.008	pse > ind > mel > pan > sin	sin vs ind sin vs pse mel vs sin pse vs pan	0.000
	Anandamide	C_22_ H_37_ N O_2_	347.28156	1.036	pse > ind > pan > mel > sin	sin vs ind sin vs pse sin vs pan	0.000
	1-Naphthaleneacetic acid	C_12_ H_10_ O_2_	186.06827	1.013	mel > ind > sin > pse > pan	mel vs pan mel vs pse mel vs sin	0.000
	Cinnamic acid	C_9_ H_8_ O_2_	148.05225	7.401	ind > pse > mel > pan > sin	sin vs ind pan vs ind sin vs pse pan vs pse mel vs sin	0.000
	neuraminic acid	C_9_ H_17_ N O_8_	267.09597	10.093	mel > ind > pan > sin > pse	mel vs pse mel vs sin pse vs ind pan vs pse	0.000
	D-Pantothenic	C_9_ H_17_ N O_5_	219.11025	5.168	mel > sin > pan > ind > pse	mel vs pse mel vs pan pse vs sin mel vs ind	0.000
Phenols and alcohols	L-Dopa	C_9_ H_11_ N O_4_	197.06852	8.41	pse > ind > pan > mel > sin	sin vs ind sin vs pan sin vs pse mel vs pse	0.000
	Paradol	C_17_ H_26_ O_3_	278.18741	1.006	ind > pse > sin > mel > pan	pan vs ind mel vs ind	0.011
	3,4-dihydroxyphenylglycol	C_8_ H_10_ O_4_	170.05758	1.012	mel > pan > ind > sin > pse	mel vs pse mel vs sin pan vs pse	0.000
	p-Cresol	C_7_ H_8_ O	108.05728	0.987	mel > sin > ind > pan > pse	mel vs pse mel vs pan mel vs ind sin vs pse	0.000
	4-Aminophenol	C_6_ H_7_ N O	109.05292	8.842	ind > mel > pan > sin > pse	pse vs ind sin vs ind pan vs ind mel vs pse mel vs pan mel vs sin	0.000
	Guaiacol	C_7_ H_8_ O_2_	124.0522	0.965	mel > pan > sin > ind > pse	mel vs pse mel vs sin mel vs ind pan vs pse	0.000
	Curcumin	C_21_ H_20_ O_6_	368.1271	4.158	mel > ind > pse > pan > sin	mel vs pse mel vs sin mel vs pan sin vs ind	0.000
	Pyridoxine	C_8_ H_11_ N O_3_	169.07316	4.818	mel > sin > pan > ind > pse	mel vs pse mel vs ind pan vs pse sin vs pse	0.000
	5-Methoxytryptophol	C_11_ H_13_ N O_2_	191.09421	10.044	mel > pan > ind > pse > sin	mel vs pse mel vs ind mel vs sin sin vs pan	0.000
	Panthenol	C_9_ H_19_ N O_4_	205.13112	11.098	pse > pan > sin > mel > ind	mel vs pse pse vs ind pan vs ind	0.000
	Metronidazole	C_6_ H_9_ N_3_ O_3_	171.06404	9.911	ind > pse > mel > pan > sin	pan vs ind sin vs ind	0.000
	Nilestriol	C_25_ H_32_ O_3_	380.23416	1.04	sin > mel > ind > pse > pan	sin vs pse sin vs ind sin vs pan mel vs pan mel vs pse	0.000
Esters	4-tert-Butylphenyl salicylate	C_17_ H_18_ O_3_	270.12098	10.232	mel > pan > sin > ind > pse	mel vs pse mel vs ind pan vs pse sin vs pse	0.000
	Tolnaftate	C_19_ H_17_ N O S	307.10486	1.028	mel > ind > pse > pan > sin	mel vs pan mel vs sin pan vs ind sin vs ind	0.000
	felbamate	C_11_ H_14_ N_2_ O_4_	238.0949	8.801	mel > ind > pan > sin > pse	mel vs pse mel vs sin pse vs ind sin vs ind	0.000
	Triethyl citrate	C_12_ H_20_ O_7_	276.12032	0.993	pse > ind > sin > pan > mel	mel vs pse	0.001
	3-O-(alpha-L-olivosyl)oleandolide	C_26_ H_44_ O10	516.29173	1.07	ind > mel > sin > pse > pan	mel vs pse mel vs pan pse vs ind pan vs ind	0.000
	epi-Tulipinolide	C_17_ H_22_ O_4_	290.15106	1.05	pse > mel > ind > sin > pan	mel vs pan mel vs sin pse vs ind pan vs pse sin vs pse	0.000
	Estriol tripropionate	C_27_ H_36_ O_6_	456.25205	0.955	sin > mel > pan > ind > pse	sin vs pse mel vs pse pan vs pse mel vs ind sin vs ind	0.000
	Promolate	C_16_ H_23_ N O_4_	293.16209	5.031	pse > ind > mel > pan > sin	sin vs pse pan vs pse	0.000
	Sirolimus	C_51_ H_79_ N O13	913.55791	7.467	pse > ind > mel > pan > sin	sin vs pse mel vs sin sin vs pan sin vs ind	0.000
Sulfur compounds	S-Methyl glutathione	C_11_ H_19_ N_3_ O_6_ S	321.09882	9.715	mel > pan > sin > ind > pse	mel vs pse mel vs ind mel vs sin sin vs pse pan vs pse	0.000
	Thiamine	C_12_ H_16_ N_4_ O S	264.10402	11.9	ind > pse > mel > pan > sin	sin vs ind sin vs pse mel vs sin sin vs ind	0.000
	Actinoquinol	C_11_ H_11_ N O_4_ S	253.04029	1.011	sin > mel > pan > ind > pse	sin vs ind sin vs pse sin vs pan	0.000
	L-Ergothioneine	C_9_ H_15_ N_3_ O_2_ S	229.08809	11.227	pan > ind > pse > mel > sin	sin vs pan mel vs pan pse vs pan	0.000
	4-Methylbenzenesulfonamide	C_7_ H_9_ N O_2_ S	171.0349	1.316	mel > ind > sin > pan > pse	mel vs pse pse vs ind sin vs pse	0.001
	2-Sulfanylbenzoic acid	C_7_ H_6_ O_2_ S	154.00852	1.007	mel > sin > ind > pan > pse	mel vs pse mel vs pan mel vs ind sin vs pse	0.000
	Promethazine sulfoxide	C_17_ H_20_ N_2_ O S	300.13167	10.929	mel > sin > pse > pan > ind	mel vs pan mel vs ind pse vs ind sin vs ind pan vs sin	0.000

*^*a*^The significantly differential metabolites were screened out by variable importance in the projection (VIP) value of PLS-DA and independent samples Kruskal–Wallis one-way analysis of variance (ANOVA) test followed by Dunn–Bonferroni *post hoc* method, with the threshold of VIP > 1.0 and corrected *P* < 0.05. ^*b*^The corrected *P-*values among the five truffle species was calculated from independent samples Kruskal–Wallis one-way analysis of variance (ANOVA) test. Abbreviations: ind, *Tuber indicum;* mel, *Tuber melanosporum;* pan, *Tuber panzhihuanense;* sin, *Tuber sinoaestivum;* pse, *Tuber pseudoexcavatum.**

Saccharides and nucleosides showed higher intensities in *T. melanosporum* (*P* < 0.05). Some metabolites, such as 2′-deoxyadenosine, *N*-acetyl-D-galactosamine, and spectinomycin, had the most amounts in *T. melanosporum*, while they exhibited significantly lower average ion intensities in *T. pseudoexcavatum* and *T. panzhihuanense* (*P* < 0.05). However, some metabolites such as hypoxanthine, nicotinamide ribotide, UDP-*N*-acetylglucosamine, 7-methylguanosine, and glucosylceramide were detected in greater amounts in *T. panzhihuanense* and *T. pseudoexcavatum* compared with the other truffles. *T. sinoaestivum* contained significantly greater amounts of adenine, adenosine, and succinyladenosine (*P* < 0.05).

Alkaloids, esters, sulfur compounds, phenols, and alcohols all showed the most strongly intensities in *T. melanosporum* (*P* < 0.05), particularly the alkaloids, including agroclavine, leonurine, betaine, piperlongumine, and sparteine. There was more sinapine and colchicine in *T. indicum* and *T. sinoaestivum*, respectively. As for phenols and alcohols, there was greater amounts of 3,4-dihydroxyphenylglycol, p-cresol, guaiacol, curcumin, pyridoxine, and 5-methoxytryptophol in *T. melanosporum.* However, paradol, 4-aminophenol, and metronidazole were more in *T. indicum.* The average ion intensity of carnitines was significantly higher in *T. indicum* than in *T. melanosporum* (*P* < 0.05), similar to that of acetyl-L-carnitine and (2E)-hexadecenoyl carnitine.

Organic acids and flavonoids exhibited the highest average ion intensities in *T. pseudoexcavatum* and *T. indicum*, with significantly lower amounts in *T. panzhihuanense* (*P* < 0.05). In terms of organic acids, there were greater amounts of mesalazine, 1-naphthaleneacetic acid, neuraminic acid, and D-pantothenic acid in *T. melanosporum*, with greater amounts of 4-pyridoxic acid and nicotinic acid in *T. indicum. T. pseudoexcavatum* contained more aspirin and (E)-ferulic acid, while *T. sinoaestivum* had significantly less anandamide and cinnamic acid. Among the few differential flavonoids, 6-methylflavone and kolaflavanone exhibited significantly higher intensity in *T. indicum*, and neobavaisoflavone was more in *T. melanosporum* and *T. panzhihuanense* compared with the other truffles.

### Metabolic Pathway Analysis Based on the KEGG Database

**FIGURE 6:**
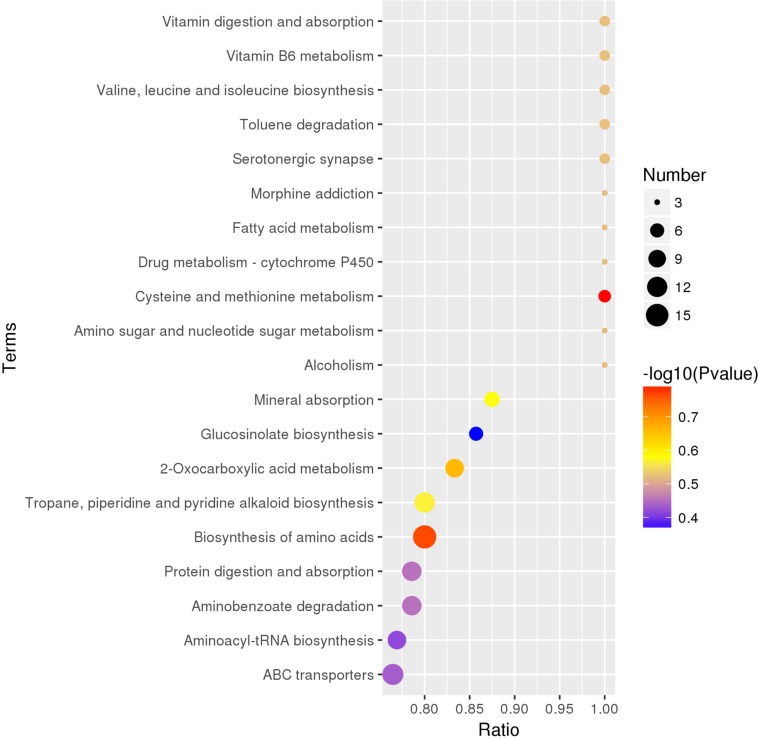
The top 20 enriched KEGG pathway terms covered by differential metabolites. On the abscissa is the ratio of the differential metabolite numbers in the corresponding pathway to the total identified metabolite numbers in this pathway, and the larger the ratio value, the higher the degree of enrichment of differential metabolites in this pathway. The color of the point represents the transformed *P*-value of the hypergeometric test, and the smaller the value, the greater the reliability and statistical significance of the test. The size of the dots represents the number of differential metabolites in the corresponding pathway, and the larger the size, the greater the number of differential metabolites within the pathway.

There were 235 identified metabolites and 149 differential metabolites annotated by the KEGG database. The differential metabolites among five truffle species covered a total of 110 pathways or metabolisms, including vitamin digestion and absorption, vitamin B6 metabolism, serotonergic synapse, drug metabolism-cytochrome P450, amino sugar and nucleotide sugar metabolism, ABC transporters, sulfur metabolism, microbial metabolism in diverse environments, biosynthesis of secondary metabolites, central carbon metabolism in cancer (Supplementary Table S1), and the 20 most enriched pathway terms are shown in the KEGG enrichment bubble diagrams (Figure 6). The content of the metabolites annotated to vitamin B6 metabolism was relatively less in *T. pseudoexcavatum* as compared to the other truffles, while the metabolites annotated to fatty acid metabolism were more in *T. pseudoexcavatum.* The metabolites annotated to the drug metabolism-cytochrome P450 and sulfur metabolism pathways were upregulated in *T. indicum* (Supplementary Figure S7a). There was higher ion intensity of annotated metabolites from cysteine and methionine metabolism in *T. melanosporum* and arachidonic acid metabolism in *T. panzhihuanense* (Supplementary Figure S7b). Many pathways, such as microbial metabolism in diverse environments, biosynthesis of secondary metabolites, mineral absorption, and biosynthesis of amino acids, involve multiple annotated metabolites, but the variation of these metabolites amounts among the five truffle species (upregulation or downregulation) was inconsistent.

## Discussion

It is important for the investigations that gather data related to the metabolites of truffle fruiting bodies, which is not only because of the unique flavor of truffles that is desired in various cuisines, but also because the knowledge regarding their nutritional value, biological activity, and the potential therapeutic usages is limited, and is in need of additional exploration (Wang and Marcone, 2011; Tang et al., 2015). With the development of technology, liquid chromatography coupled to mass spectrometry approach can be widely applied in metabolomics studies currently, having a wide detected range and the high specificity and sensitivity (Velez et al., 2007). In the current study, this method was used to analyze the metabolic profiling of truffle fruiting bodies of five species (*T. indicum*, *T. melanosporum*, *T. panzhihuanense*, *T. sinoaestivum*, and *T. pseudoexcavatum*), which showed satisfactory data quality. Except for *T. melanosporum* and *T. indicum*, previous studies on the metabolites of the other three truffle species have been rare.

The results in this study revealed that there were significant differences in the metabolic characteristics among the five truffle species, especially between *T. melanosporum* and the other four truffles. The difference between *T. indicum* and *T. pseudoexcavatum* was relatively small. Besides the genetic reasons, combined with the different host plants and different soil properties around the ascocarps from these different truffle species, it should be because these truffles have different ecological, nutritional, climatic and geographical needs. In other studies, it was found that the metabolite composition was complex, and the metabolite accumulation was variable because it was greatly affected by genetic and environmental factors (Bertault et al., 1998; Mannina et al., 2012; Splivallo et al., 2012; Tang et al., 2017), such as growth characteristics, development stage, preservation conditions, and geographical origins. In this study, we detected the metabolites in the reproductive stage of truffles, and the mature degree and preservation conditions of truffle fruiting bodies were basically the same, however, the different geographical location led the significant difference of their ecology, like the vegetation (host plants) and soil we had investigated. The host plants of *T. melanosporum* belongs to *Quercus* while the other four truffles’ host plants are *Pinus;* and *T. melanosporum* grow in the alkaline soil while the other truffles in this study adapted to slightly acidic soils, which showed the obvious differences of the ecological environment between *T. melanosporum* and the other four truffles. This should be closely related to the significant differences in the metabolic characteristics between *T. melanosporum* and the other four truffles. In terms of other soil properties which all showed the obvious differences, the basic nutrient status of the soil associated with *T. indicum* was relatively good overall and the soil nutrients of *T. panzhihuanense* were poor compared with the soils around the other truffles. These soil characteristics may also related to the variation of metabolites in truffle fruiting bodies, which need further exploration of the specific effects. In previous studies, Diaz et al. (2003) compared the aroma profiles of *T. melanosporum* and *T. aestivum* at different geographical zones, and indicated that different truffle aroma metabolites could result from a difference in both truffle species and geographical areas of origin, which had the similarity with our study. From the perspective of phylogenetic analysis of these five truffle species in this study, *T. indicum* was phylogenetically related to *T. melanosporum* (Zhang et al., 2019), and *T. sinoaestivum* had relatively closer phylogenetical relationship with *T. melanosporum* and *T. indicum* compared with the other two truffle species, but their metabolic profiling was clearly different from each other. Previous researches said that the diverse ecological environment may cause a high level of genetic variability, which finally led to the obvious difference in metabolites (Bertault et al., 1998). Except for above factors, the effect of microbial communities on the metabolites variation in truffle fruiting bodies could not be ignored, because truffle fruiting bodies harbor a diverse microbes, including bacteria and fungi, etc., and they play an important role in the truffle ascocarps formation as well as their aroma (Antony-Babu et al., 2014; Vahdatzadeh et al., 2015). So the metabolites produced by microbes were also included in truffle fruiting bodies, which could not been seen as a single organism to investigate. Meanwhile, the species of truffle, the soil properties and host plants all had the influence on the microbial communities (Vahdatzadeh et al., 2015; Benucci and Bonito, 2016; Ye et al., 2018), which may finally lead the differences of metabolic profiling in truffle fruiting bodies. So the research on the truffle-associated microbiome and the related metabolites from these microbiome was an important step in future for further exploring the metabolic mechanism of truffles. And the associated microbiome may play an important role of bridge for the correlation between truffle metabolic profiling and the specific ecological environment or growth stage.

As for the detected metabolites, although thetotal ion intensity of all the identified metabolites was different in the five truffles, which was higher in *T. melanosporum*, the types of these metabolites were similar. A total of 1282 metabolites with significantly differential amounts among the five truffle species were detected, accounting for approximately 54% of the 2376 identified metabolites, which covered various chemical classes including amino acids, saccharides, nucleosides and their analogs, organic acids, alkaloids, flavonoids, carnitines, amides, ketones, phenols and alcohols, esters, sulfur compounds, and others. Although previous studies also detected these chemical categories in other truffle species (Wang and Marcone, 2011; El Enshasy et al., 2013; Patel et al., 2017), few mentioned on the nucleosides and their analogs, alkaloids, carnitines, or flavonoids in the truffle fruiting bodies.

As was reported, nucleosides and their analogs exhibited widely varied biochemical activities, such as antiviral and anticonvulsant activity, and maintaining the immune response (Ballarin et al., 1987; Liu et al., 2011). Adenine, adenosine, 2′-deoxyadenosine, 7-methylguanosine, and 5′-S-methyl-5′-thioadenosine were detected in our study, as well as in previous studies (Liu et al., 2011; Longo et al., 2017), and the average content of saccharides, nucleosides, and their analogs was significantly higher in *T. melanosporum*, *T. sinoaestivum*, and *T. panzhihuanense*. Adenine is an electron-rich substance known to bind to electrical ions and interfere with oxidative protection (Goncçalves et al., 2010), and their amounts were greater in *T. sinoaestivum* and *T. melanosporum.*

Alkaloids from herbs exhibited wide biological and pharmacological activities (Fang et al., 2011), and had been identified in these five truffles, with a significantly greater amounts in *T. melanosporum*. Many of the alkaloids had antioxidant, anti-tumor, anti-inflammatory, or antidepressant-like effects, such as colchicine, sinapine, leonurine, and piperlongumine (Guo et al., 2016; Kumar et al., 2016; Jia et al., 2017; Karki et al., 2017), which were predominant in *T. sinoaestivum, T. indicum*, and *T. melanosporum*. The number of differential carnitines and flavonoids was fewer, and in *T. indicum*, their ion intensity was higher. Acetyl-L-carnitine, a powerful antioxidant that can easily penetrate the blood–brain barrier to the central nervous system, has an obvious protective effect on nerves (Ferreira and McKenna, 2017). There was significantly more of it in *T. indicum* and *T. panzhihuanense.* Neobavaisoflavone is also a bioactive compound that was isolated from *Psoralea corylifolia*, and possesses significant anti-inflammatory and anti-cancer functions (Szliszka et al., 2011). Great amounts of it were found in *T. melanosporum* and *T. panzhihuanense.*

As was demonstrated, among the aromatic metabolites, aldehydes and alcohols were the two most abundant aromatic components in truffles, while the sulfur-containing compounds such as dimethyl sulfide and dimethyl disulfide were considered the key components to human sensory interpretation (Splivallo et al., 2007; Splivallo and Ebeler, 2015). Esters, sulfur compounds, phenols, and alcohols are the metabolites involved in truffle aroma which received much attention in previous researches (Vahdatzadeh et al., 2015), and were also detailly described in our study. Consistently, they were all more abundant in *T. melanosporum.* Many phenolic compounds, such as paradol, *p*-cresol, 4-aminophenol, guaiacol, and curcumin, exhibit bioactivities, having anti-inflammation, antioxidation, antimicrobial, and antitumoral characteristics (Villares et al., 2012; Lee and Lee, 2014; Gaire et al., 2015), and most of them were found in greater amounts in *T. melanosporum* and *T. indicum*. Another study (Patel et al., 2017) showed that *T. melanosporum* contained anandamide, a fatty acid neurotransmitter that is an immunomodulator of the central nervous system and was found to have the ability to lead in the process of cancer cell apoptosis (Patsos et al., 2010; Patel et al., 2017). In our study, this metabolite existed in the all five truffle species, but with significantly greater amounts in *T. sinoaestivum.* Wang and Marcone summarized the nutritional content variation in different truffle species, such as *T. magnatum, T. borchii*, and *T. aestivum* (Wang and Marcone, 2011), and similarly, the five truffle species in our study also differed in their nutritional content, e.g., amino acids, although the truffle species we used was different from this previous study.

The metabolic pathways of the metabolites were also analyzed according to the KEGG database, which reflected the most important biochemical metabolic pathway and signal transduction pathway involved by metabolites that had differential amounts patterns. Truffles produced metabolic molecules during each stage of their growth for necessary survival, and the metabolites from truffles change during their life cycle and also during the maturity stage of the truffles (Streiblova et al., 2012; Mello et al., 2013). To research their metabolism mechanisms, it would be helpful to roundly explore the formation mechanism of truffle ascocarps. At present, it is found that the truffle formation is the result of the sexual reproduction of the fungi, and the stages of the development of *T. melanosporum* are described in previous studies, but some parts of their life cycle are also missing (Murat et al., 2013; Le Tacon et al., 2016). A previous report examined the metabolic profiles of the ectomycorrhizae of *T. indicum*, showing the metabolic pathways of *T. indicum* ectomycorrhizae, which include linoleic acid metabolism, and dalanine, aspartate, and glutamate metabolism (Li et al., 2018). However, the metabolic pathways of truffle fruiting bodies in our study were obviously different from those. In this study, a total of 110 pathways or metabolisms were covered by the differential metabolites among five truffle species. The most enriched pathway terms were vitamin digestion and absorption, vitamin B6 metabolism, and valine, leucine, and isoleucine biosynthesis. However, most metabolic reactions involved multiple metabolites, and the variation of these metabolites amounts among the five truffle species was inconsistent. Therefore, it cannot be simply said that the expression of some metabolic pathways was increased or decreased in a certain truffle species, which indicates that there were complex changes in the metabolic mechanisms in truffle fruiting bodies that require our further investigation.

In terms of the methods in this study, using untargeted analysis, metabolomics can be used to simultaneously detect and quantify a wide range of compounds that cover various chemical classes, which is ideal for the detection of unexpected changes or unknown information in metabolite levels (Bloszies and Fiehn, 2018; Gomez-Gomez et al., 2019). Also in this study, a lot of amounts of the metabolites were found in different truffle species, which would broaden the knowledge of metabolites of different truffle species in different environments. Meanwhile, the LC-MS approach also contributes to a better result due to its intrinsic selectivity, specificity and sensitivity (Gomez-Gomez et al., 2019). In the future research about truffles, untargeted metabolomics can also be considered to use, such as investigating the metabolic variations in different stage of truffles, under different storage conditions, in a specific environment, or in the fermentation process. Additionally, the genomic and transcriptomics data can be connected to the construction of genome metabolic network of truffles, which would be helpful to systematically understand truffles and realize the truffles artificial cultivation. However, the technical challenges of this approach are also exist, such as filtering procedures improvement for untargeted LC-MS metabolomics data (Schiffman et al., 2019), completing the functional database for metabolomics research.

In conclusion, through the LC-MS-based metabolomics method, the significantly different metabolic profiles of these five commercial truffle species were obtained, with the biggest difference between *T. melanosporum* and the other four truffles, which should be the results of comprehensive effects of genetic factors and different ecology, including different truffle species, host plants, and soil characteristics. The amounts of many metabolites were different in five truffles and every truffle species had their predominantly accumulated metabolites. The chemical categories of the differential metabolites were rich among the five truffle species, which covered a total of 110 pathways or metabolisms. Each of the nine categories with detailed description in this study all contained some metabolites with bioactivities that are beneficial to human beings, like acetyl-L-carnitine, neobavaisoflavone, anandamide. Generally, compared with the other four truffles, there were more compounds with higher intensity in *T. melanosporum*, which indicated that *T. melanosporum* has a higher value for utilization as a drug or food, and that it is reasonable for it to command a higher price. Besides, the maturity, the life cycle, and the associated microbes all had the effects on the metabolic profiles of different truffle species, which is interesting to combine these for the further exploration, aiming to better reveal the metabolic mechanism of truffles and may enhance the insight into the truffle research.

## Data Availability Statement

The datasets generated for this study can be found in the NCBI database with GenBank accession numbers MN338092–MN338096.

## Author Contributions

XL, XZ, and BZ conceived and designed the experiments. LY, ZK, DJ, and LFY performed the experiments. XZ and XL wrote and revised the manuscript. All authors approved the final version of the manuscript.

## Conflict of Interest

The authors declare that the research was conducted in the absence of any commercial or financial relationships that could be construed as a potential conflict of interest.
