# Supplementary material for: LC-MS-Based Metabolomic Approach Revealed the Significantly Different Metabolic Profiles of Five Commercial Truffle Species
Source: Front Microbiol. 2019 Sep 25;10:2227. doi: 10.3389/fmicb.2019.02227 (PMC6773953; doi:10.3389/fmicb.2019.02227)
Supplement: Supplementary file 1 [file Table_1.DOCX]

Supplementary Material

# Supplementary Figures and Tables

## Supplementary Figures


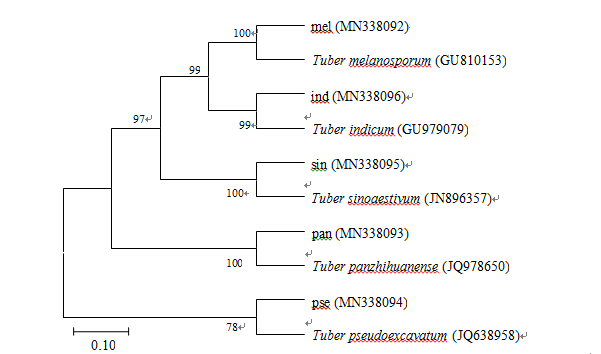


**Supplementary Figure 1.** The phylogenetic tree based on ITS genes sequences using Neighbor-joining. Branch numbers represent the support rate of self-development. Scales represent the difference of nucleotide bases, which is 0.10. The number of random sampling calculations is 1000.


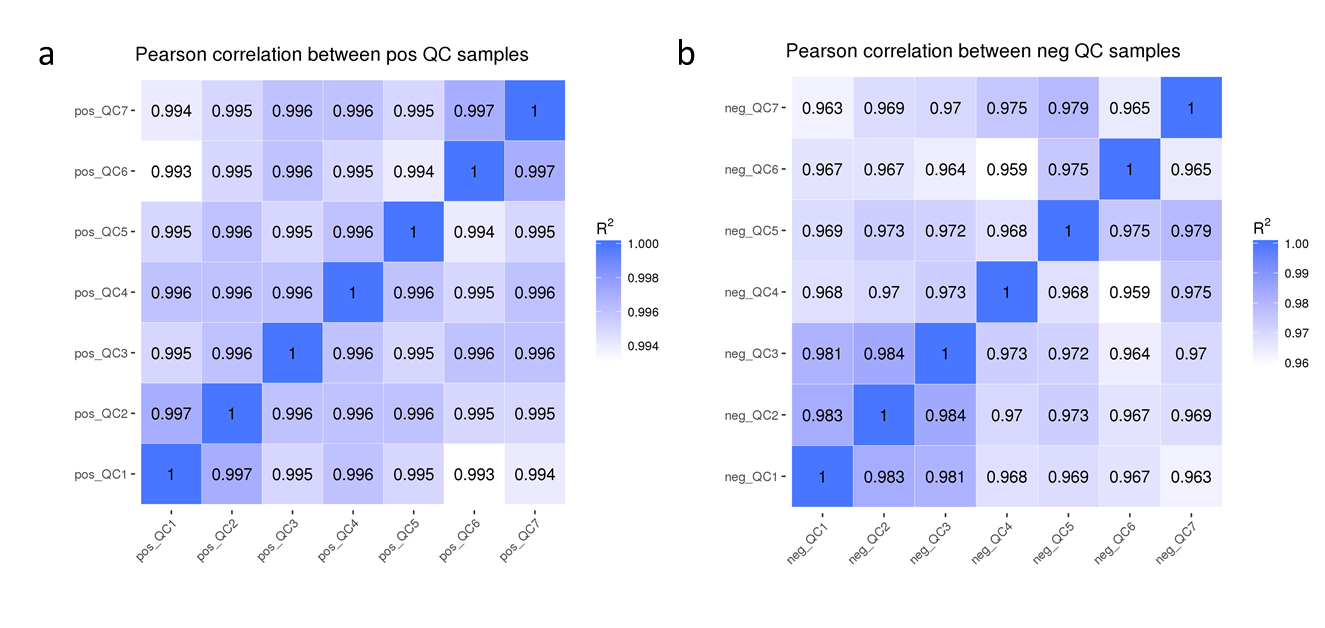
**Supplementary Figure 2.** The Pearson correlation analyse between QC (Quality control) samples in both positive (a) and negative (b) ion modes

**
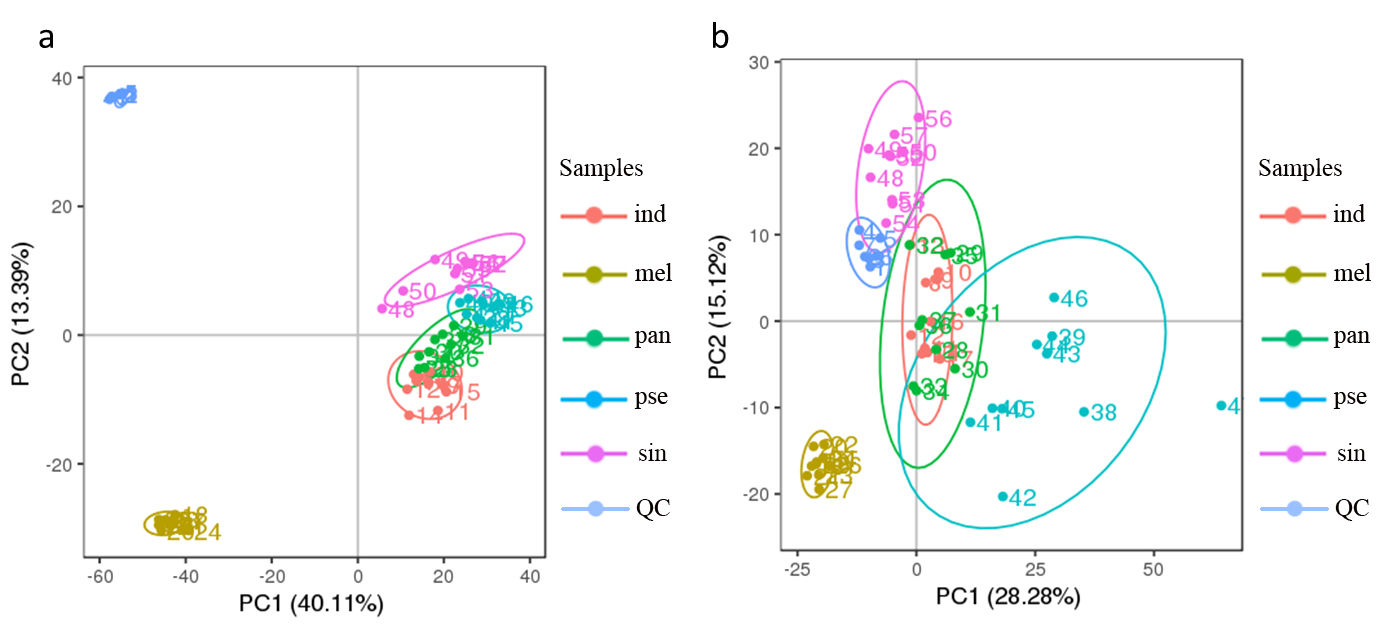
Supplementary Figure 3.** The PCA (Principal component analysis score) plots of all the samples in both positive (a) and negative (b) ion modes. Abbreviations: ind, *Tuber indicum*; mel, *Tuber melanosporum*; pan, *Tuber panzhihuanense*; sin, *Tuber sinoaestivum*; pse, *Tuber pseudoexcavatum*.


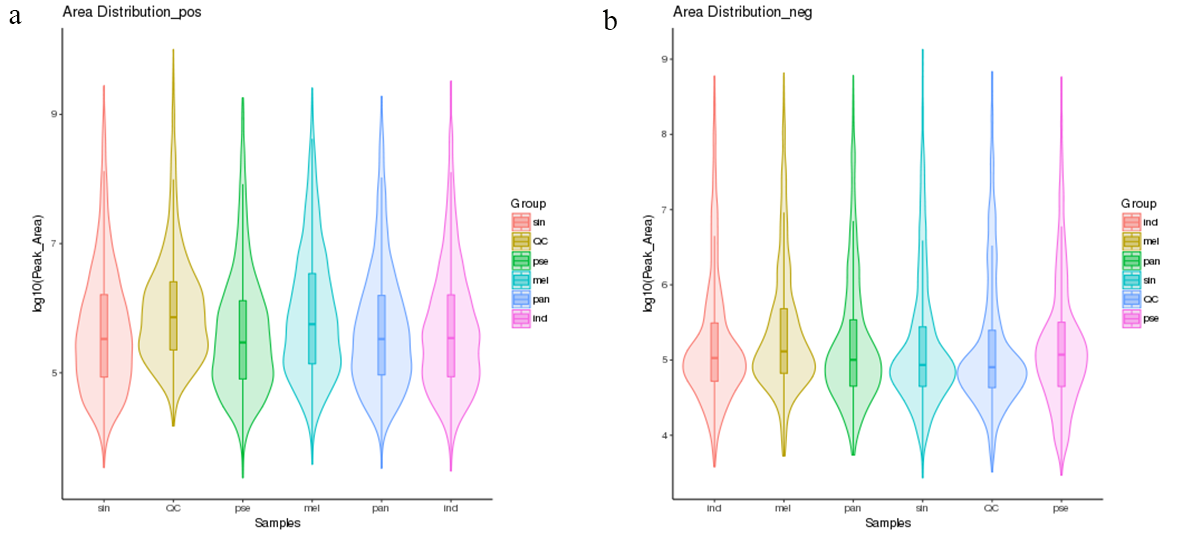


**Supplementary Figure 4.** The violin plots that show the distribution of metabolite ion intensity in different sample groups in both positive (a) and negative (b) ion modes. Sample groups are on the abscissa, and the transformed values of the average peak areas of metabolites in each sample group are on the ordinate. Abbreviations: ind, *Tuber indicum*; mel, *Tuber melanosporum*; pan, *Tuber panzhihuanense*; sin, *Tuber sinoaestivum*; pse, *Tuber pseudoexcavatum*.


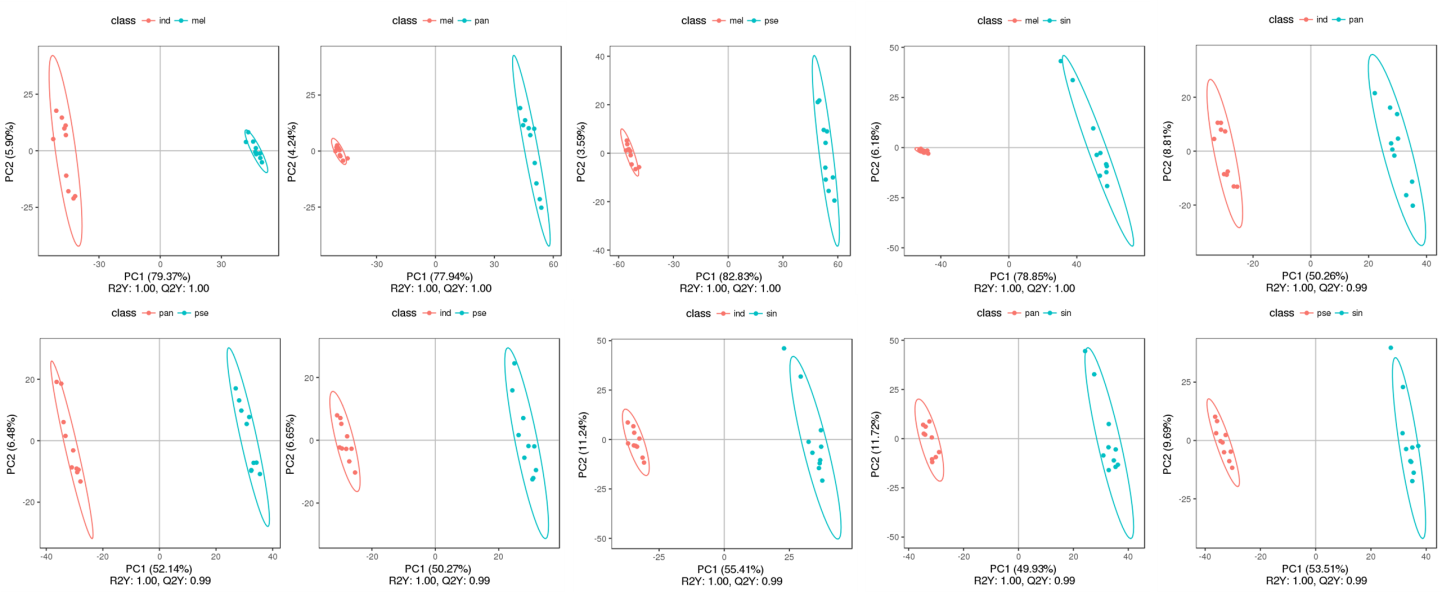


**Supplementary Figure 5.** The PLS-DA (partial least squares-discriminant analysis) score plots of pairwise comparation among five truffle species in positive ion mode. Abbreviations: ind, *Tuber indicum*; mel, *Tuber melanosporum*; pan, *Tuber panzhihuanense*; sin, *Tuber sinoaestivum*; pse, *Tuber pseudoexcavatum*.


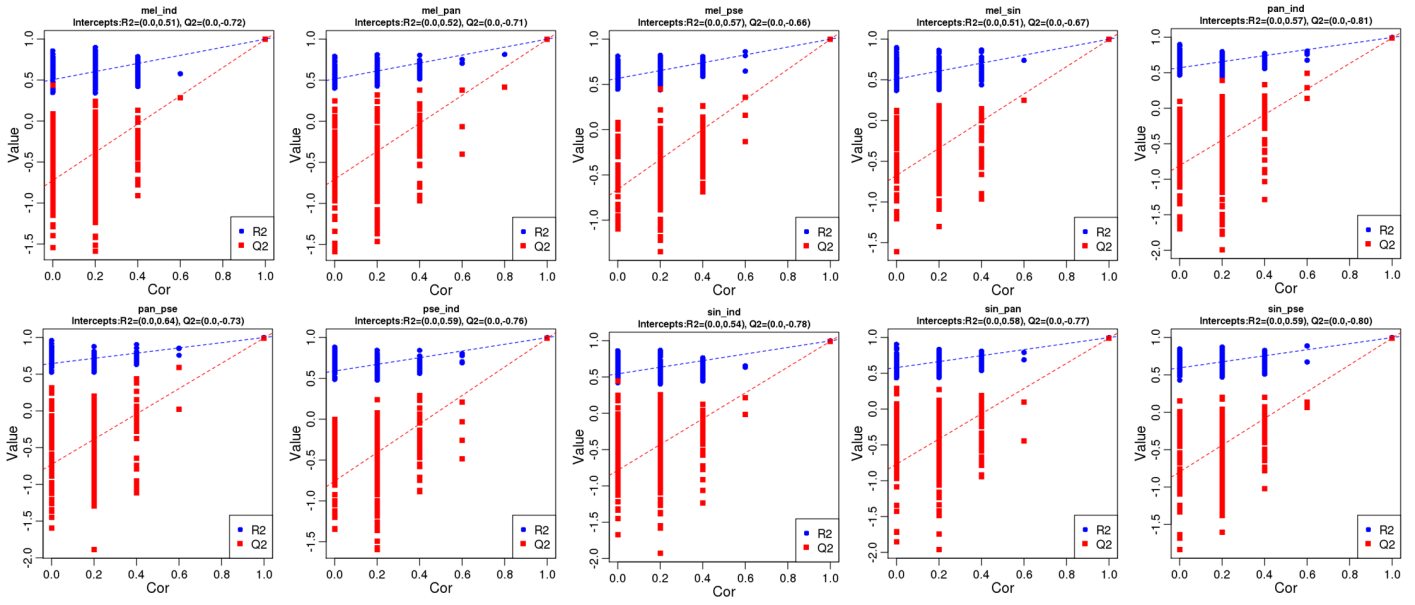


**Supplementary Figure 6.** The validation of PLS-DA (partial least squares-discriminant analysis) models of pairwise comparation among five truffle species in positive ion mode. Abbreviations: ind, *Tuber indicum*; mel, *Tuber melanosporum*; pan, *Tuber panzhihuanense*; sin, *Tuber sinoaestivum*; pse, *Tuber pseudoexcavatum*.


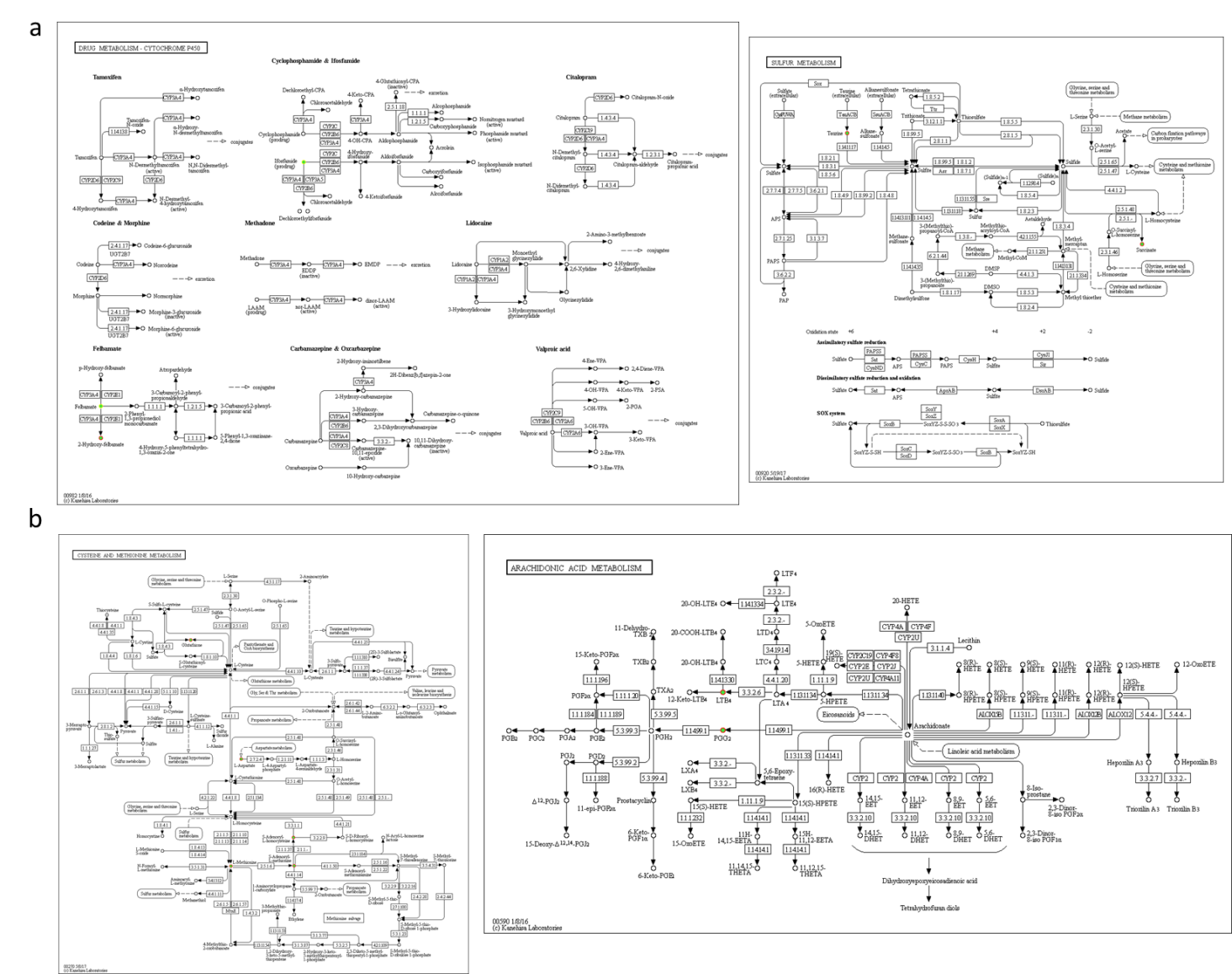


**Supplementary Figure 7.** KEGG maps of (a) Drug metabolism-cytochrome P450 and sulfur metabolism; (b) Cysteine and methionine metabolism and arachidonic acid metabolism

## Supplementary Tables

**Supplementary Table 1.** The metabolic pathways covered by all the differential metabolites.

| MapTitle | P value | Adjusted  Pv | x1 | x2 | n | N | Enrich  Direct |
| --- | --- | --- | --- | --- | --- | --- | --- |
| Cysteine and methionine metabolism | 0.1613236 | 1 | 5 | 5 | 149 | 235 | Over |
| Biosynthesis of amino acids | 0.1712558 | 1 | 16 | 20 | 149 | 235 | Over |
| 2-Oxocarboxylic acid metabolism | 0.2189374 | 1 | 10 | 12 | 149 | 235 | Over |
| Mineral absorption | 0.2637666 | 1 | 7 | 8 | 149 | 235 | Over |
| Tropane, piperidine and pyridine alkaloid biosynthesis | 0.2704235 | 1 | 12 | 15 | 149 | 235 | Over |
| Valine, leucine and isoleucine biosynthesis | 0.299479 | 1 | 4 | 4 | 149 | 235 | Over |
| Toluene degradation | 0.299479 | 1 | 4 | 4 | 149 | 235 | Over |
| Vitamin B6 metabolism | 0.299479 | 1 | 4 | 4 | 149 | 235 | Over |
| Serotonergic synapse | 0.299479 | 1 | 4 | 4 | 149 | 235 | Over |
| Vitamin digestion and absorption | 0.299479 | 1 | 4 | 4 | 149 | 235 | Over |
| Amino sugar and nucleotide sugar metabolism | 0.3009274 | 1 | 3 | 3 | 149 | 235 | Over |
| Drug metabolism - cytochrome P450 | 0.3009274 | 1 | 3 | 3 | 149 | 235 | Over |
| Fatty acid metabolism | 0.3009274 | 1 | 3 | 3 | 149 | 235 | Over |
| Morphine addiction | 0.3009274 | 1 | 3 | 3 | 149 | 235 | Over |
| Alcoholism | 0.3009274 | 1 | 3 | 3 | 149 | 235 | Over |
| Aminobenzoate degradation | 0.3529886 | 1 | 11 | 14 | 149 | 235 | Over |
| Protein digestion and absorption | 0.3529886 | 1 | 11 | 14 | 149 | 235 | Over |
| ABC transporters | 0.3682141 | 1 | 13 | 17 | 149 | 235 | Over |
| Aminoacyl-tRNA biosynthesis | 0.3834928 | 1 | 10 | 13 | 149 | 235 | Over |
| Glucosinolate biosynthesis | 0.4274719 | 1 | 6 | 7 | 149 | 235 | Over |
| Primary bile acid biosynthesis | 0.5339516 | 1 | 2 | 2 | 149 | 235 | Over |
| Valine, leucine and isoleucine degradation | 0.5339516 | 1 | 2 | 2 | 149 | 235 | Over |
| Benzoxazinoid biosynthesis | 0.5339516 | 1 | 2 | 2 | 149 | 235 | Over |
| Arachidonic acid metabolism | 0.5339516 | 1 | 2 | 2 | 149 | 235 | Over |
| Nitrotoluene degradation | 0.5339516 | 1 | 2 | 2 | 149 | 235 | Over |
| Sulfur metabolism | 0.5339516 | 1 | 2 | 2 | 149 | 235 | Over |
| Biosynthesis of unsaturated fatty acids | 0.5339516 | 1 | 2 | 2 | 149 | 235 | Over |
| Vancomycin resistance | 0.5339516 | 1 | 2 | 2 | 149 | 235 | Over |
| Sulfur relay system | 0.5339516 | 1 | 2 | 2 | 149 | 235 | Over |
| Longevity regulating pathway - worm | 0.5339516 | 1 | 2 | 2 | 149 | 235 | Over |
| Retrograde endocannabinoid signaling | 0.5339516 | 1 | 2 | 2 | 149 | 235 | Over |
| GABAergic synapse | 0.5339516 | 1 | 2 | 2 | 149 | 235 | Over |
| Cocaine addiction | 0.5339516 | 1 | 2 | 2 | 149 | 235 | Over |
| Amphetamine addiction | 0.5339516 | 1 | 2 | 2 | 149 | 235 | Over |
| Nicotine addiction | 0.5339516 | 1 | 2 | 2 | 149 | 235 | Over |
| Pertussis | 0.5339516 | 1 | 2 | 2 | 149 | 235 | Over |
| African trypanosomiasis | 0.5339516 | 1 | 2 | 2 | 149 | 235 | Over |
| Eicosanoids | 0.5339516 | 1 | 2 | 2 | 149 | 235 | Over |
| Purine metabolism | 0.5431825 | 1 | 9 | 12 | 149 | 235 | Over |
| Tryptophan metabolism | 0.5836252 | 1 | 11 | 15 | 149 | 235 | Over |
| Bile secretion | 0.5836252 | 1 | 11 | 15 | 149 | 235 | Over |
| Lysine degradation | 0.6549015 | 1 | 4 | 5 | 149 | 235 | Over |
| Pantothenate and CoA biosynthesis | 0.6549015 | 1 | 4 | 5 | 149 | 235 | Over |
| Parkinson's disease | 0.6549015 | 1 | 4 | 5 | 149 | 235 | Over |
| beta-Alanine metabolism | 0.7138241 | 1 | 6 | 8 | 149 | 235 | Over |
| Nicotinate and nicotinamide metabolism | 0.7138241 | 1 | 6 | 8 | 149 | 235 | Over |
| Phenylalanine metabolism | 0.7499824 | 1 | 7 | 10 | 149 | 235 | Over |
| Biosynthesis of plant hormones | 0.7729964 | 1 | 9 | 13 | 149 | 235 | Over |
| Biosynthesis of alkaloids derived from ornithine, lysine and nicotinic acid | 0.8218765 | 1 | 13 | 19 | 149 | 235 | Over |
| Microbial metabolism in diverse environments | 0.885516 | 1 | 32 | 49 | 149 | 235 | Over |
| Metabolic pathways | 0.9949257 | 1 | 69 | 108 | 149 | 235 | Over |
| Biosynthesis of phenylpropanoids | 1 | 1 | 10 | 15 | 149 | 235 | Over |
| Biosynthesis of antibiotics | 1 | 1 | 25 | 39 | 149 | 235 | Over |
| Biosynthesis of secondary metabolites | 1 | 1 | 41 | 64 | 149 | 235 | Over |
| Naphthalene degradation | 1 | 1 | 2 | 3 | 149 | 235 | Over |
| Folate biosynthesis | 1 | 1 | 2 | 3 | 149 | 235 | Over |
| Metabolism of xenobiotics by cytochrome P450 | 1 | 1 | 2 | 3 | 149 | 235 | Over |
| Quorum sensing | 1 | 1 | 2 | 3 | 149 | 235 | Over |
| Plant hormone signal transduction | 1 | 1 | 2 | 3 | 149 | 235 | Over |
| Dopaminergic synapse | 1 | 1 | 2 | 3 | 149 | 235 | Over |
| Pentose phosphate pathway | 1 | 1 | 1 | 1 | 149 | 235 | Over |
| Fructose and mannose metabolism | 1 | 1 | 1 | 1 | 149 | 235 | Over |
| Fatty acid degradation | 1 | 1 | 1 | 1 | 149 | 235 | Over |
| Secondary bile acid biosynthesis | 1 | 1 | 1 | 1 | 149 | 235 | Over |
| Arginine biosynthesis | 1 | 1 | 4 | 6 | 149 | 235 | Over |
| Monobactam biosynthesis | 1 | 1 | 3 | 4 | 149 | 235 | Over |
| Lysine biosynthesis | 1 | 1 | 3 | 4 | 149 | 235 | Over |
| Penicillin and cephalosporin biosynthesis | 1 | 1 | 1 | 1 | 149 | 235 | Over |
| Arginine and proline metabolism | 1 | 1 | 7 | 11 | 149 | 235 | Over |
| Clavulanic acid biosynthesis | 1 | 1 | 1 | 1 | 149 | 235 | Over |
| Phenylalanine, tyrosine and tryptophan biosynthesis | 1 | 1 | 4 | 6 | 149 | 235 | Over |
| Indole diterpene alkaloid biosynthesis | 1 | 1 | 1 | 1 | 149 | 235 | Over |
| Phenazine biosynthesis | 1 | 1 | 1 | 1 | 149 | 235 | Over |
| Cyanoamino acid metabolism | 1 | 1 | 6 | 9 | 149 | 235 | Over |
| N-Glycan biosynthesis | 1 | 1 | 1 | 1 | 149 | 235 | Over |
| Lipopolysaccharide biosynthesis | 1 | 1 | 1 | 1 | 149 | 235 | Over |
| Peptidoglycan biosynthesis | 1 | 1 | 1 | 1 | 149 | 235 | Over |
| Pyruvate metabolism | 1 | 1 | 1 | 1 | 149 | 235 | Over |
| Propanoate metabolism | 1 | 1 | 1 | 1 | 149 | 235 | Over |
| Styrene degradation | 1 | 1 | 1 | 1 | 149 | 235 | Over |
| Carbon fixation in photosynthetic organisms | 1 | 1 | 1 | 1 | 149 | 235 | Over |
| Biotin metabolism | 1 | 1 | 1 | 1 | 149 | 235 | Over |
| Zeatin biosynthesis | 1 | 1 | 3 | 4 | 149 | 235 | Over |
| Phenylpropanoid biosynthesis | 1 | 1 | 6 | 9 | 149 | 235 | Over |
| Isoflavonoid biosynthesis | 1 | 1 | 1 | 1 | 149 | 235 | Over |
| Stilbenoid, diarylheptanoid and gingerol biosynthesis | 1 | 1 | 1 | 1 | 149 | 235 | Over |
| Steroid degradation | 1 | 1 | 1 | 1 | 149 | 235 | Over |
| Biosynthesis of ansamycins | 1 | 1 | 1 | 1 | 149 | 235 | Over |
| Acridone alkaloid biosynthesis | 1 | 1 | 1 | 1 | 149 | 235 | Over |
| Endocrine resistance | 1 | 1 | 1 | 1 | 149 | 235 | Over |
| PPAR signaling pathway | 1 | 1 | 1 | 1 | 149 | 235 | Over |
| cAMP signaling pathway | 1 | 1 | 5 | 7 | 149 | 235 | Over |
| Sphingolipid signaling pathway | 1 | 1 | 1 | 1 | 149 | 235 | Over |
| Neuroactive ligand-receptor interaction | 1 | 1 | 8 | 12 | 149 | 235 | Over |
| Ferroptosis | 1 | 1 | 1 | 1 | 149 | 235 | Over |
| Cellular senescence | 1 | 1 | 1 | 1 | 149 | 235 | Over |
| Synaptic vesicle cycle | 1 | 1 | 4 | 6 | 149 | 235 | Over |
| Glutamatergic synapse | 1 | 1 | 1 | 1 | 149 | 235 | Over |
| Regulation of actin cytoskeleton | 1 | 1 | 1 | 1 | 149 | 235 | Over |
| Estrogen signaling pathway | 1 | 1 | 1 | 1 | 149 | 235 | Over |
| Proximal tubule bicarbonate reclamation | 1 | 1 | 1 | 1 | 149 | 235 | Over |
| Pancreatic secretion | 1 | 1 | 1 | 1 | 149 | 235 | Over |
| Cholesterol metabolism | 1 | 1 | 1 | 1 | 149 | 235 | Over |
| Central carbon metabolism in cancer | 1 | 1 | 5 | 7 | 149 | 235 | Over |
| Penicillins | 1 | 1 | 1 | 1 | 149 | 235 | Over |
| Benzoic acid family | 1 | 1 | 1 | 1 | 149 | 235 | Over |
| Serotonin receptor agonists/antagonists | 1 | 1 | 1 | 1 | 149 | 235 | Over |
| Catecholamine transferase inhibitors | 1 | 1 | 1 | 1 | 149 | 235 | Over |
| Potassium channel blocking and opening drugs | 1 | 1 | 1 | 1 | 149 | 235 | Over |
| N-Metyl-D-aspartic acid receptor antagonists | 1 | 1 | 1 | 1 | 149 | 235 | Over |

MapTitle, the name of enriched KEGG Pathway; P value, the original p value calculated from the enrichment analysis; Adjusted Pv, the adjusted p value; x1, the number of the differential metabolites associated with this pathway; x2, the number of all the metabolites (background) associated with this pathway; n, the number of the differential metabolites annotated by KEGG; N, the number of all the metabolites (background) annotated by KEGG; Enrich Direct: enrichment points, “Over” stands for enrichment.
